# Patterns of Pathomorphological Changes in Acute Necrotizing Pancreatitis

**DOI:** 10.1155/2012/508915

**Published:** 2012-04-30

**Authors:** I. Kovalska, O. Dronov, S. Zemskov, E. Deneka, M. Zemskova

**Affiliations:** ^1^Department of General Surgery No. 1, Bogomolets National Medical University, Kyiv 03039, Ukraine; ^2^Department of Pathology, Kyiv City Hospital No. 10, Kyiv 03039, Ukraine; ^3^Kyiv Center for Liver, Bile Ducts and Pancreas Surgery, Kyiv 03039, Ukraine

## Abstract

Acinar necrosis is the basic microscopic sign of acute necrotizing pancreatitis (ANP). Microcirculation disorder is one of the major factors in the pathogenesis and morphogenesis of ANP besides free radicals and damage of enzymatic origin. This study is dedicated to the description of microscopic changes in the pancreatic stroma in ANP, which leads to destruction of the exocrine pancreas with a putative mechanism of endocrine function preservation. This study has been carried out on histological samples of pancreas from 224 patients with ANP. Histological staining was performed with hematoxylin-eosin (H&E), Masson, Gomori methods, and PAS. Microscopy was performed with magnifications of 40×, 100×, and 400×. Vascular endothelial desquamation, stasis, and sludge are typical changes in microcirculation observed in early stages of ANP. Initially, parietal circular intravascular microthrombosis accompanied by endothelial desquamation as early as stromal swelling occurs with no detectable necrosis. Residual stroma appears between areas of necrosis and intact pancreatic tissue. Mucoid swelling is first seen in the perivascular spaces extending to the parenchyma and changing into fibrinoid imbibition causing further necrosis. Reticulin argyrophilic backbone surrounding the pancreatic acini and small ducts decompose. Pancreatic structures, which may be preserved in necrotic tissue, include nerves, major ducts, and Langerhans islets.

## 1. Introduction

Necrosis and tissue degradation are the basic microscopic signs of acute necrotizing pancreatitis (ANP). These changes can be focal or diffuse. We believe that the microcirculation disorder is one of the strongest factors in the pathogenesis and morphogenesis of ANP besides free radicals and of enzymatic origin.

## 2. Aim

This study is dedicated to investigation and description of microscopic changes in the pancreas in ANP which leads to the destruction of the exocrine pancreas, changes in the pancreatic stroma, and a presumable mechanism of preservation of endocrine function.

## 3. Materials and Methods

This study has been carried out on histological samples of pancreas from 224 patients with ANP. All these patients were operated at the Kyiv Center for Hepatopancreatobiliary Surgery (named after V. S. Zemskov) from 1997 through 2004. Postmortem examinations of autopsy tissue were performed in 21 of the 224 patients. The samples were investigated at varying times compared to the onset of the disease. 82.5% of the patients presented with alimentary and 17.5% with biliary pancreatitis. The study of the histologic tissues was routinely performed by utilizing hemotoxylin-eosin, Masson, Gomori, and PAS stains. Microscopy was performed at 40×, 100×, and 400×  magnification.

## 4. Results and Discussion

The pictures of a pancreatic disorder in ANP are very diverse.

**Figure 1:**
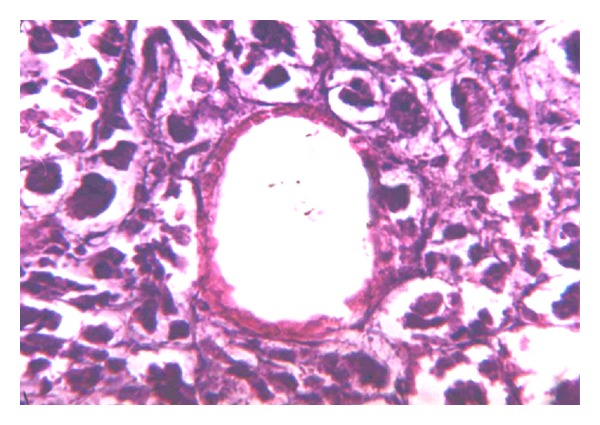
Interstitial swelling of pancreatic parenchyma. Microcirculation peripheral thrombosis. H&E (×400).

**Figure 2:**
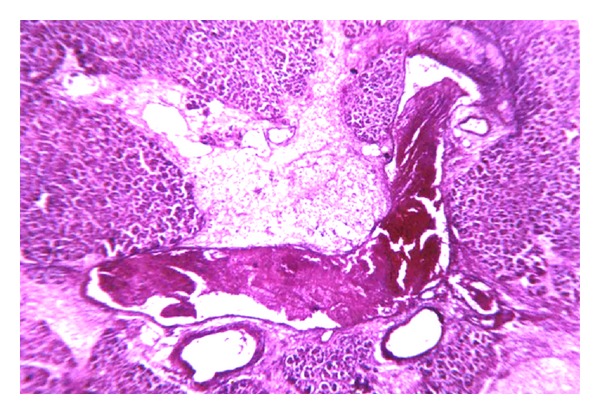
Focal necrosis of pancreatic parenchyma associated with massive thrombosis in venules. H&E (×40).

Vascular endothelial desquamation, stasis, and sludge are typical changes that are seen in the microcirculation that occurs in early stages of ANP and which subsequently evolves into thrombosis. We believe that thrombosis of the pancreatic microcirculation is one of the leading factors in the course of ANP. This hypothesis correlates with recent data of other investigators [1–4]. Initially, intravascular microthrombosis are seen as parietal circular changes (Figure 1) accompanied by endothelial desquamation. These changes are detected in the early stages when stromal swelling appears and there is no necrosis as yet. Apparently parietal circular thrombosis does not cause complete arrest of the circulation. That is why the first minimal signs of necrosis appear after thrombosis progresses into total obstruction of the vessels (Figure 2).

Masson's trichrome staining is the histochemical method of choice for investigation of thrombotic morphogenesis.

**Figure 3:**
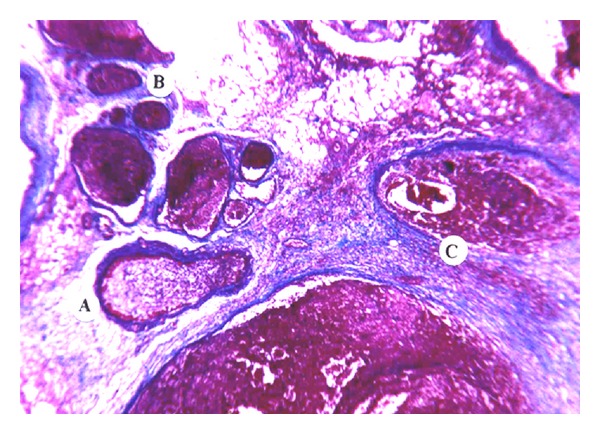
Sequence of thrombus formation: parietal thrombus progressing centripetally (A); total thrombosis (B); vessel remodeling (C). Masson staining (×40).

Thrombi start appearing at the periphery and extend towards the center and appear as an internal “death mask” of the vessel. Thereafter, fibrin masses grow at the periphery of the thrombus as a gracile net, then shrinkage and cicatrization follow. Thrombolysis or recanalization can frequently be seen together with the above-mentioned changes in a single sample or even in a single microscopic field (Figure 3).

**Figure 4:**
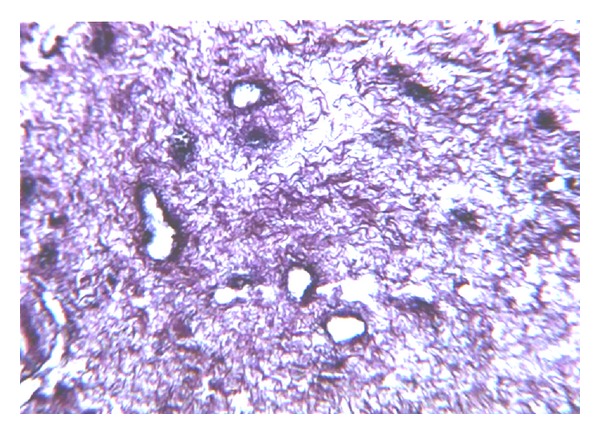
Argyrophilic fibers are preserved only around major structures on the background of necrotic changes in the pancreas. Gomori staining (×100).

In normal pancreatic tissue a thin black net is clearly seen surrounding each acinar cell or cell clusters. This net reflecting the shape of an acinus is the reticulin argyrophilic backbone. The brown coarse fibrotic folds that divide the reticulin net are collagen. The changes in the focus of necrosis affect, first of all, the reticulin argyrophilic backbone. In ANP the gracile structure of the argyrophilic reticulin carcass disappears and foci of decomposition are seen as brown structureless fibrotic components surrounding intact vessels or acinar ducts (Figure 4).

**Figure 5:**
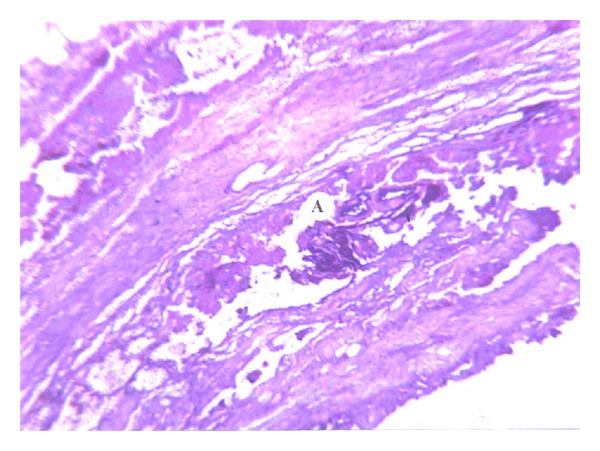
PAS-positive substance accumulation (A) in zones of fibrinoid necrosis. PAS reaction for neutral mucopolysaccharides (×100).

Mucoid changes of the stromal carcass which at first are reversible, progress and become visible with routine hematoxylin-eosin staining. Most often mucoid swelling is located in perivascular spaces, but it may also extend and change into fibrinoid imbibition. Fibrin and fibrinoids are easily detected by Masson staining wherein fibrinoids are seen as a bright red and fibrin as a dark red or claret color that shows a fasciculated structure. Intact connective tissue appears as blue or cyan fibers. Foci with fibrinoid changes progress into necrosis and assume PAS-positive features. When stained by Shiff reagent they turn into deep crimson color (Figure 5). Later these foci may accumulate calcium and that is why calcinosis found in pancreatic parenchyma may indicate earlier ANP.

**Figure 6:**
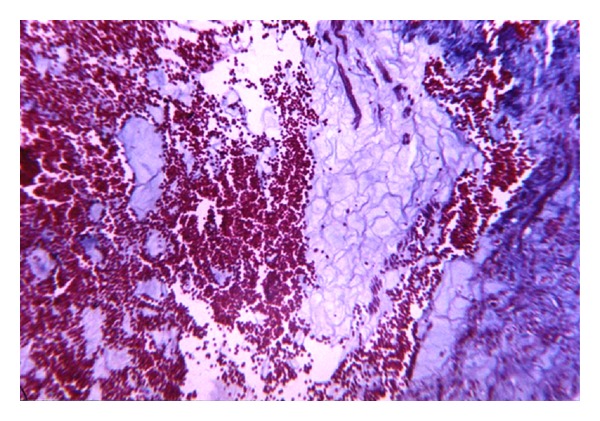
Fresh massive diapedesis (hemorrhages) in pancreas with ANP. Masson staining (×100).

As microcirculatory paresis persists and vascular permeability increases, the mucoid swelling is complicated by hemorrhage. In ANP, hemorrhages in the pancreatic parenchyma are an obligatory event and their manifestation varies widely from minimal to massive. The mechanism of these hemorrhages is considered diapedesis (Figure 6). Extravasated red blood cells undergo lysis and the extracellular hemoglobin consequently degrades resulting in catabolic hemosiderin formation in the pancreatic parenchyma.

Stromal components arise between the necrotic hemorrhages and the intact pancreatic tissue. Early parenchymal changes in ANP are usually discrete and are probably associated with certain stromal features even though the stroma itself is not clearly distinguishable in any given sample.

**Figure 7:**
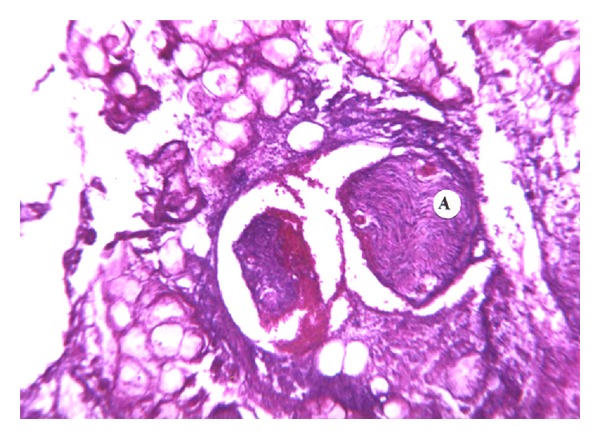
Neural fibers resistant to necrotic changes (A). H&E (×100).

In ANP, nerves are resistant to destruction (Figure 7). Nerve structure is well preserved and nerve function is presumably intact in fields of total necrosis. This fact may explain the persistent pain syndrome in ANP patients.

**Figure 8:**
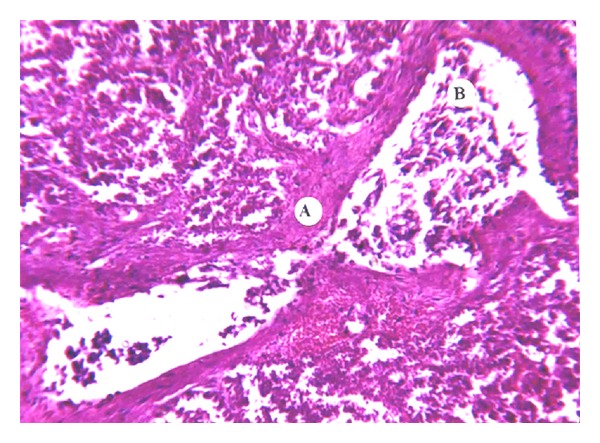
Pancreatic ducts resistant to necrotic changes (A). Desquamation of ductal epithelium (B). H&E (×100).

Amidst acinar necrosis, lobular ducts appear to be relatively stable structures (Figure 8). Their resistance to necrosis is apparently proportional to their diameter. However, in all our findings, there was evidence of desquamation of ductal epithelium in samples from ANP patients.

Microscopic changes in the islets of Langerhans, in ANP patients, are also of great interest.

**Figure 9:**
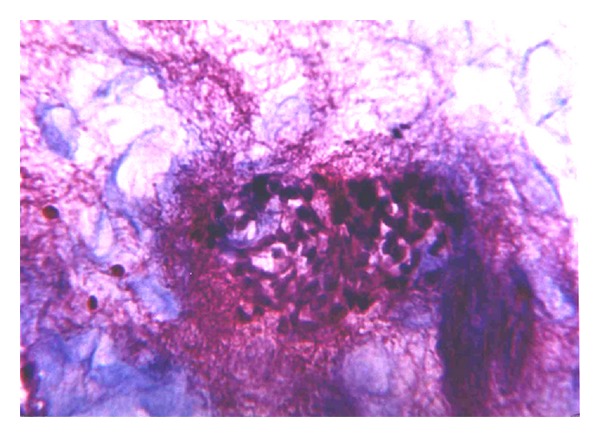
Islets of Langerhans resistant to necrotic changes (A). H&E (×400).

**Figure 10:**
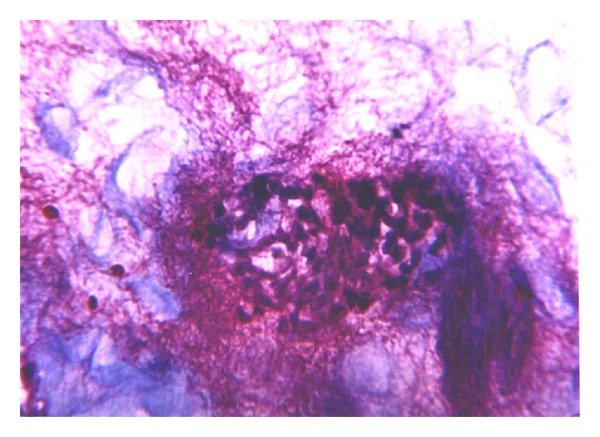
Fibrin masses noted around Langerhans islet in the zone of necrosis. Masson staining (×100).

It is well known that in follow-up studies of ANP patients, only about 10–26% develop pancreatogenic diabetes after recovery [5, 6]. Diabetes is not necessarily present in patients with total exocrine pancreatic insufficiency. Microscopically, the structure of the islets of Langerhans is intact among masses of detritus (Figure 9). When stained with Masson, the islets of Langerhans are frequently surrounded by a gracile net of fibrin (Figure 10) which shows a deep claret color. Hence we may assume that this formation of a fibrin net around the islet of Langerhans contributes to its resistance and preservation of its structure in the aggressive microenvironment in patients with ANP.

Table 1 represents quantitative distribution of the microscopic changes in pancreas among patients with APN.

In conclusion, our study correlates with recently published data [1–4] and underlines the importance and supremacy of early pancreatic microcirculation and local coagulation disorders (44.2 to 100%) in the pathogenesis of ANP. Our study shows that despite aggressive surrounding necroses the islets of Langerhans are preserved in 74.1% of cases, most probably due to fibrin capsule formation. Therefore, surgical removal of necrotic tissue should be performed with maximal prudence and tissue preservation in order to decrease the risk of pancreatic endocrine insufficiency in patients with ANP. On admission it may be advisable to use anticoagulation therapy in the early stages of acute pancreatitis, irrespective of the severity of the disease, in order to minimize the extent of pancreatic tissue ischemia and necrosis.

## Figures and Tables

**Table 1:** Early, late, and protective changes in pancreatic tissue in patients with APN.

Microscopic changes	Number of	Percentage
	patients	
*Early changes*		
Vascular endothelial desquamation	224	100%
Intravascular stasis and sludge	215	95.9%
Circular thrombosis	99	44.2%
Mucoid swelling in stromal carcass	40	17.9%
Parenchymal hemorrhages	208	92.9%
*Late changes*		
Parenchymal necrosis	224	100%
Total thromboses	206	92%
Thrombolysis and recanalization	49	21.9%
Decomposition of argyrophilic	211	94.2%
reticular carcass		
Fibrinoid swelling	164	73.2%
Calcium accumulation	34	15.2%
*Protective changes*		
Nerves resistant to necrosis	179	79.9%
Lobular ducts resistant to necrosis	175	78.1%
Islets of Langerhans resistant to	166	74.1%
necrosis		

## References

[1] Uhlmann D, Lauer H, Serr F, Witzigmann H (2008). Pathophysiological role of platelets and platelet system in acute pancreatitis. *Microvascular Research*.

[2] Cuthbertson CM, Christophi C (2006). Disturbances of the microcirculation in acute pancreatitis. *British Journal of Surgery*.

[3] Hackert T, Pfeil D, Hartwig W (2007). Platelet function in acute experimental pancreatitis. *Journal of Gastrointestinal Surgery*.

[4] Kerner T, Vollmar B, Menger MD, Waldner H, Messmer K (1996). Determinants of pancreatic microcirculation in acute pancreatitis in rats. *Journal of Surgical Research*.

[5] Malecka-Panas E, Gasiorowska A, Kropiwnicka A, Zlobinska A, Drzewoski J (2002). Endocrine pancreatic function in patients after acute pancreatitis. *Hepato-gastroenterology*.

[6] Burge MR, Gabaldon-Bates J (2003). The role of ethnicity in post-pancreatitis diabetes mellitus. *Diabetes Technology and Therapeutics*.

